# Subclinical Hypothyroidism in Perimenopausal Abnormal Uterine Bleeding Patients

**DOI:** 10.7759/cureus.21839

**Published:** 2022-02-02

**Authors:** Ali Sebtain, Muhammad Qasim, Abroo Bahadur, Amjad Ali, Kashif A Samin, Moiz Ahmed

**Affiliations:** 1 Department of Endocrinology, Hayatabad Medical Complex (HMC), Peshawar, PAK; 2 Department of Medicine, Mian Rashid Hussain Shaheed Memorial Hospital, Nowshera, PAK; 3 Department of Medicine, Bacha Khan Medical College, Mardan, PAK; 4 Department of Family Medicine, Khyber Medical University, Peshawar, PAK; 5 Department of Cardiology, National Institute of Cardiovascular Diseases, Karachi, PAK

**Keywords:** subclinical hypothyroidism, perimenopausal, menopause, hypothyroidism, abnormal uterine bleeding

## Abstract

Background

Abnormal uterine bleeding (AUB) can be very troublesome and is common in women with thyroid dysfunction. The current study aimed to assess the incidence of subclinical hypothyroidism in women with perimenopausal AUB.

Methodology

A cross-sectional study was conducted at Hayatabad Medical Complex (HMC), Peshawar, Pakistan, between September 2020 to February 2021. All outdoor female patients with complications in the obstetrics and gynecology department, aged between 40 to 55 years of age, and no obvious cervical and genital lesions were included in the study. Patients with a history of suspected inflammatory disease, use of oral contraceptives, and malignant lesions of the cervix were excluded from the study. All cases were evaluated for AUB and their thyroid profile was evaluated. Data regarding menstrual irregularities were recorded in a pre-defined proforma and clinical examination was performed.

Results

A total of 500 women were enrolled with a mean age of 47.2 ± 7.3 years. Of these, 234 (46.8%) women were overweight and the mean levels of the thyroid-stimulating hormone were 4.4 ± 2.5 mIU/L. The mean triiodothyronine and thyroxine were 3.2 ± 1.9 and 1.5 ± 0.7 pmol/L, respectively. The rate of subclinical hypothyroidism was 33%. It was shown that the body mass index was significantly correlated with subclinical hypothyroidism (p=0.03). Furthermore, the rate of oligomenorrhea was significantly higher in patients with subclinical hypothyroidism (p=0.05).

Conclusion

This study highlights the association between thyroid dysfunction in women with menstrual disorders. Screening and surveillance of thyroid-related abnormalities are warranted in patients with menstrual irregularities to avoid complications of the disease.

## Introduction

Perimenopausal abnormal uterine bleeding (AUB) is defined as uterine blood flow that is erratic [1]. This is one of the most prevalent presentations in gynecology clinics where patients experience blood flow that does not meet the typical bleeding characteristics such as volume, occurrence, duration, and/or cycles [1,2].

AUB is a frequently occurring disorder that affects 15-20% of women between the phases of early puberty and menopause. AUB may present as sexual malfunction, irregular menstrual cycle, and early menopause. The beginning of clinically overt hypothyroidism or hyperthyroidism is anticipated by menstrual disturbances [2,3].

Among women with hypothyroidism, hyperprolactinemia is induced by thyrotropin-releasing hormone (TRH). This stimulation modifies the pulsation of the Gonadotropin-releasing hormone (GnRH). The alteration results in anovulation and luteal phase deficiencies due to hindered response of luteinizing hormone (LH) [4]. Thyroid hormones also regulate the interaction of the follicle-stimulating hormone (FSH) and LH receptors; an effect that is significantly important in the production of progesterone hormone. Hypothyroidism also lowers the synthesis of sex hormone-binding globulin (SHBG) and affects the peripheral consumption of estrogen. The abnormal activity results in aberrant pituitary feedback [5,6]. Moreover, it also interrupts the production of coagulation factors such as Factor VII, VIII, IX, and XI, thereby instigating menorrhagia. In cases of hyperthyroidism, the production of SHBG increases substantially. The synthesis of estrogens from androgens in the periphery is augmented, and estrogen metabolism is disrupted [7].

Correspondingly, the prevalence of subclinical hypothyroidism (SCH) is 5-8% among adult women, and it increases up to 20% by the age of 60 years. It is characterized as the thyroid-stimulating hormone (TSH) levels less than 10 mIU/L [8]. One of the primary indicators of subchorionic hemorrhage (SCH) is menorrhagia and the patients are at higher risk of developing elevated lipid profile and anemia. These effects result in the development of coronary artery disease, pregnancy complications, and miscarriages [9].

There exists a strong relationship between the thyroid hormone and regular steroid activity. The activity is crucial for the ovaries to function and therefore impacts fertility. The clinical management of thyroid dysfunction is essential to improve fertility and regulate the menstrual cycle [10,11]. The presence of menstrual abnormalities, sterility, and comorbidities are common among women with disrupted thyroid function. This study was undertaken to assess the relationship between perimenopausal abnormal uterine bleeding and subclinical hypothyroidism.

## Materials and methods

A cross-sectional study was conducted at the Department of Endocrinology and Diabetes, Hayatabad Medical Complex (HMC) Peshawar, Pakistan, between September 2020 to February 2021. A non-probability, consecutive sampling technique was used to recruit participants in the study. Ethical approval was obtained from the Institutional Review Board (IRB) committee of HMC (Reference # IRB/289134/HMC). 

The sample size was determined using OpenEpi. By keeping the prevalence of subclinical hypothyroidism as 43.52% [12], the margin of error of 4.33%, and the confidence level of 95%, the calculated sample size was 500. All outdoor female patients with complications in the obstetrics and gynecology department, aged between 40-55 years of age, and no obvious cervical and genital lesions were included in the study. Patients with suspected inflammatory disease, women on oral contraceptives, those with premalignant and malignant lesions of the cervix, and intrauterine contraceptive device users were excluded from the study.

Informed consent was obtained prior to the enrolment of patients in the study. Each woman was evaluated for abnormal uterine bleeding and the thyroid profile was evaluated. The study protocol included a thorough history taking regarding menstrual irregularities using a predesigned and prestructured proforma. The clinical examination, which included general physical examination, neck examination, and systemic and gynecological examinations, was carried out. Routine blood investigations like hemoglobin, erythrocyte sedimentation rate, liver function test, random blood sugar, bleeding time, clotting time, pap smear, endometrial biopsy, transabdominal and transvaginal ultrasonography, serum triiodothyronine (T3), thyroxine (T4), and TSH were also done.

Patient data was compiled and analyzed through IBM SPSS Statistics for Windows, Version 21.0 (Released 2012. IBM Corp., Armonk, New York). Frequency and percentage were computed for levels of BMI, thyroid status, type of bleeding pattern. Mean ± SD was calculated for age, weight, height, BMI, serum levels TSH, T3, and T4. Stratification was done on age, levels of BMI, thyroid status, type of bleeding pattern to see the effect of these modifiers on the outcome using the chi-square test. A p-value < 0.05 was considered statistically significant.

## Results

The mean age of patients was 47.2 ± 7.3 years. The majority of the women were overweight with a frequency of 234 (46.8%). The mean levels of the TSH were 4.4 ± 2.5 mIU/L while the levels for triiodothyronine and thyroxine were 3.2 ± 1.9 and 1.5 ± 0.7 pmol/L, respectively (Table 1). Subclinical hypothyroidism was found in 165 (33%) females. 

**Table 1:** The sociodemographic characteristics of the participants

Characteristics	Mean ± SD / n (%)
Mean age (years)	47.2 ± 7.3
40-50 years	354 (70.8%)
>50 years	146 (29.2%)
Weight (kg)	67.8 ± 9.6
Height (m)	1.52 ± 1.01
BMI (kg/m^2^)	28.6 ± 5.7
BMI groups	
Underweight	2 (0.4%)
Normal	84 (16.8%)
Overweight	233 (46.8%)
Obese	181 (36.2%)
Thyroid-stimulating hormone level (mIU/L)	4.4 ± 2.5
Triiodothyronine level (pmol/L)	3.2 ± 1.9
Thyroxine level (pmol/L)	1.5 ± 0.7
Types of bleeding pattern	
Menorrhagia	238 (47.6%)
Metrorrhagia	67 (13.4%)
Oligomenorrhea	144 (28.8%)
Hypomenorrhea	51 (10.2%)
Thyroid status	
Overt hypothyroidism	133 (26.6%)
Subclinical hypothyroidism	165 (33%)
Euthyroid	202 (40.4%)

Table 2 illustrates the relationship between patient characteristics and subclinical hypothyroidism. About 65% of women between the ages of 40-50 years had subclinical hypothyroidism; however, age was not significantly associated with the condition (p=0.11). The study showed that the BMI was significantly associated with subclinical hypothyroidism (p=0.03). Furthermore, the rate of oligomenorrhea was significantly higher in patients with subclinical hypothyroidism (p=0.05). 

**Table 2:** Stratification of demographics against subclinical hypothyroidism

Characteristics	Subclinical hypothyroidism		p-value
	Positive	Negative	
Age groups			0.11
40-50 years	106 (64.24%)	247 (73.9%)	
>50 years	59 (35.76%)	87 (26.1%)	
BMI groups			0.03
Underweight	0 (0%)	2 (0.6%)	
Normal	38 (22.9%)	46 (13.7%)	
Overweight	86 (51.8%)	148 (44.2%)	
Obese	42 (25.3%)	139 (41.5%)	
Types of bleeding pattern			0.05
Under menorrhagia	72 (42.6%)	165 (50%)	
Metrorrhagia	32 (18.9%)	34 (10.3%)	
Oligomenorrhea	55 (32.5%)	89 (27%)	
Hypomenorrhea	10 (5.9%)	42 (12.7%)	

## Discussion

According to our findings, the majority of the women with AUB had normal thyroid levels however almost 30% had subclinical hypothyroidism. Subclinical hypothyroidism was significantly associated with BMI and oligomenorrhea. These findings are in agreement with previous studies authored by Mohapatra et al. [13], and Parveen et al. [14].

Most women in the present study were overweight and presented with menorrhagia. However, women with subclinical hypothyroidism frequently presented with oligomenorrhea. The findings are inconsistent with a previous study by George et al., who revealed that about 46.42% of women in their study presented with menorrhagia. Of these, 41% had thyroid disorders and subclinical hypothyroidism constituted about 17.86% of the disorders [15]. Similarly, another study by Singh et al. revealed that almost three-fourths of hypothyroid women had menorrhagia [16]. In support of the present study, Pushpagiri et al. revealed that in a cohort of 300 infertile women, 27% had hypothyroidism while 25% had subclinical hypothyroidism [17].

According to Kolli et al., in patients with subclinical and overt hypothyroidism, menorrhagia was found to be 55% prevalent, followed by polymenorrhagia, acyclical bleeding, polymenorrhea, oligomenorrhea, and metrorrhagia [18]. In another study conducted among women of reproductive age, subclinical hypothyroidism was found in about 15 seemingly normal individuals with AUB (15%) [19]. Pujari et al. conducted a study in a tertiary hospital in India on patients with subclinical hypothyroidism. It was observed that 86.7% of the selected subjects experienced substantially heavy bleeding and 37.5% suffered from the irregular menstrual cycle [20]. A study by Byna et al. was consistent with our study as it reported that out of the 55 patients presented with AUB, 12 (21.8%) had hypothyroidism while seven (12.7%) had hyperthyroidism [21]. Meanwhile, in our study, 133 (26.6%) patients had overt hypothyroidism and 165 (33%) patients had subclinical hypothyroidism. 

The result from this study reflects a significant association of AUB with thyroid dysfunction. However, several limitations deserve to be highlighted. The participants were recruited from a single tertiary health care facility and the study did not include subjects from diverse backgrounds. The generalizability is, therefore, not possible to other facilities and participants from different environments. Nonetheless, the findings are consistent with previous research and underline the significance of routine screening of AUB patients for thyroid disorders.

## Conclusions

This study concludes that menstrual irregularities are significantly more common in patients with hyperthyroidism followed by hypothyroidism. Hence, thyroid dysfunction should be considered an important etiological factor for menstrual abnormalities. Screening and surveillance of thyroid-related abnormalities are warranted in patients with menstrual irregularities to avoid complications of the disease.
